# Comparing case-control study for treatment of proximal tibia fractures with a complete metaphyseal component in two centers with different distinct strategies: fixation with Ilizarov frame or locking plates

**DOI:** 10.1186/s13018-018-0792-3

**Published:** 2018-05-22

**Authors:** Haakon Berven, Michael Brix, Kaywan Izadpanah, Eva Johanna Kubosch, Hagen Schmal

**Affiliations:** 1 Department of Orthopaedics and Traumatology, Odense University Hospital, Sdr. Boulevard 29, 5000 Odense C, Odense, Denmark; 2 Department of Clinical Research, University of Southern Denmark, Odense, Denmark; 3 Department of Orthopedics and Trauma Surgery, Medical Center, Albert-Ludwigs-University of Freiburg, Faculty of Medicine, Albert-Ludwigs-University of Freiburg, Freiburg, Germany

**Keywords:** Proximal tibia fracture, External fixation, Locking plates, Ilizarov, Open reduction and internal fixation, Complete metaphyseal fracture

## Abstract

**Background:**

The purpose of this study was to compare two methods of stabilization for proximal tibia fractures (AO 41) with a complete metaphyseal component, external fixation with the Ilizarov wire frame, and internal fixation with locking plates.

**Methods:**

Patients from two level 1 trauma centers treated between 2009 and 2015 were included in a retrospective comparing cohort study. The first center stabilized the non-pathological, proximal tibia fractures exclusively with external fixation and the second with internal plating. Combined clinically and radiologically evaluated, bone healing was the primary outcome. The secondary outcomes included complications, range of motion (ROM) and axial alignment of the knee, the reoperation rate within 6 months, heterotopic ossifications (HTO), and signs of posttraumatic osteoarthritis (PTOA). A logistic regression analysis corrected for uneven distributed parameters.

**Results:**

The 62 patients treated with Ilizarov frame and the 68 patients treated with plate fixation were comparable regarding epidemiological parameters, injury characteristics, and comorbidity except for injury severity score (ISS) and smoking behavior. The time of healing was shorter in the group undergoing plate fixation (*p* = 0.041); however, the incidence of non-unions was equal. Furthermore, there was no difference regarding the rate of deep infections, thrombosis, alignment, reoperations, PTOA, and ROM. Heterotopic ossifications were more prevalent following plate fixation (13.2 vs 1.6%, *p* = .013). External fixation was associated with a higher rate of superficial infections (40.4 vs 2.9%, *p* = .000). The initial displacement, the incidence of deep infections, and the classification significantly influenced the incidence of non-unions in both groups (*p* < 0.02).

**Conclusions:**

Fixation of proximal tibia fractures with plates resulted in a slightly shorter healing time compared to Ilizarov frame stabilization. Furthermore, the complication profiles differ with more heterotopic ossifications and less superficial infections following internal plating.

**Trial registration:**

DRKS, DRKS00013275, Registered 11/2/2017, Retrospectively registered.

**Electronic supplementary material:**

The online version of this article (10.1186/s13018-018-0792-3) contains supplementary material, which is available to authorized users.

## Background

The concurrent presence of a joint and a metaphyseal component is a typical biomechanical characteristic of proximal tibia fractures, which more often result in mal-unions than more distally located fractures [1]. Indeed, only 5–11% of these fractures have no joint involvement, when the metaphysis is completely interrupted [2]. Whereas the articular surface requires anatomical reduction and internal fixation providing absolute stability, the metaphysis is usually fixed with implants offering relative stability considering the local biological requirements [3]. Locking plates, which were introduced in the 1990s [4], allow an effective treatment addressing both parts. A more traditional approach is the combination of lag screws for joint reconstruction with an Ilizarov frame stabilizing the metaphysis. Clinical results were reported for both treatment standards [5, 6]; however, comparison of studies are lacking. Recently, a systematic review evaluated both principals based on the published cohort studies [7]. Although the article makes a differentiated analysis available, there are obvious difficulties. The included studies report mainly case series and do not compare both techniques. Furthermore, inclusion criteria or outcome parameter do not match each other. Overall, the evidence of current literature is limited. In conclusion, there is a need for comparative studies. A major problem in orthopedic surgery is that the procedure under investigation needs to be carried out with a specific competence. Therefore, multicenter study designs with a “one arm – one center”––design experienced a growing popularity, because they allow that a specific operation is performed repeatedly and with high quality in one of the participating hospitals [8].

The study aim was a comparison of two treatment strategies for proximal tibia fractures with a complete metaphyseal component, the stabilization using an Ilizarov frame or locking plates. Each operating method is a standard procedure in one of the participating centers. The primary endpoint is the degree of bony consolidation, which is clinically important and well documented. The secondary endpoints were the quality of healing and complications. Our hypothesis was that there would be comparable healing for the two treatment methods and a different complication profile.

## Methods

### Patient identification and injury characteristics

Patients with proximal tibia fractures treated by external fixation with the Ilizarov wire frame were identified by searching patient records in the COSMIC electronic medical journal system (Cambio, Odense, Denmark) and the Accident Research Group database of the Odense University Hospital, Denmark. The diagnosis code DS821 for proximal tibia fractures and the procedure code KNGJ21 for external fixation were used (SKS-browser). The PROMetheus electronic medical journal system fracture of the Freiburg University Hospital, Germany (PROMetheus, Klinikrechenzentrum Freiburg, Germany), allowed to identify patients treated with plate fixation with the ICD diagnosis code S82.1 for proximal tibia fractures. Patients operated between 2009 and 2015 were included in the study.

The radiographs and patient records from the resulting list were then assessed for inclusion and exclusion criteria. Inclusion criteria were patients with an AO 41 A2, A3, or C1–C3 fracture, who were treated either with external fixation using the Ilizarov wire frame or open reduction and internal fixation using locking plates. Exclusion criteria were patients younger than 15 years of age at the time of the fracture or with open growth plates, patients with definitive operative fixation of the fracture more than 3 weeks after trauma, and patients with pathological fractures (including osteoporotic fractures). The follow-up period was 6 months because this allowed defining the presence of a non-union. If bony consolidation was clear clinically and radiologically documented after 3 months without later complications, the status was used for analysis. Fractures were classified using standard antero-posterior and lateral conventional radiographs. Available CT scans, standard diagnostics in both contributing centers, supplemented the assessment. Age, gender, smoking behavior, and body mass index (BMI) defined the epidemiological characteristics of the populations, the ASA score (physical status according to the American Society of Anesthesiologists), and the comorbidity. The discrimination between the epiphyseal (AO 41) and the diaphyseal part (AO 42) of the tibia was facilitated by a square whose sides had the same length as the widest part of the epiphysis according to the AO classification of long bones [9]. Fractures were further categorized as either closed or open. The quality of documentation did not allow discriminating safely between different grades, for which reason this simplified classification was used. The Injury Severity Score (ISS) was calculated for each patient based on admittance and discharge records [10]. The trauma mechanism was classified as high energy, when a fall from a significant height above 1.5 m, a traffic accident, crush damage from heavy falling objects, or high-speed sport injuries were described. Otherwise, the accident was evaluated as a low-energy trauma, correlating with simple falls. The injury severity was further categorized as monotrauma, when only the proximal tibia was significantly injured, as multiple injured, when more injuries were found, but the ISS was < 16, and as polytrauma when the ISS was ≥ 16. All fractures in both centers were operated by experienced consultants specialized in fracture treatment.

### Primary outcome

The primary outcome was bone union. The method employed was described by Cole et al. 2004 [11] and includes combined clinical symptoms and radiological signs indicating bony consolidation such as callus formation and the absence of considerable pain during full weight bearing on the fractured extremity. Union was recorded as occurring either within the first 3 months or between 3 and 6 months. Non-union was defined as lacking evidence of union after 6 months [6]. In one case, a fracture had been recorded as healed after 6 months; however, later, a non-union was documented and treated. This was also evaluated as a non-union.

### Secondary outcomes

The type of infection was classified into two categories: superficial and deep. Superficial infections involved only the skin and subcutaneous tissue. Any event during the therapy requiring some kind of treatment such as antibiotics indicated an infection. The presence of a deep infection was assumed, when a septic arthritis and/or osteomyelitis was documented requiring i.v. antibiotics and operative irrigation with revision or removal of the fixating implants.

A compartment syndrome or a peroneal nerve paresis was registered pre- and post-operatively, indicating a severe soft tissue damage. The displacement of fracture fragments was assessed by the initial radiographs and was defined as the maximal distance between two components out of both the antero-posterior and lateral radiographs.

The posttraumatic osteoarthritis was evaluated using the Kellgren-Lawrence scale [12]. The range of motion of the knee joint was assessed for all patients. When the journal records documented a normal status, the extension/flexion was assumed to be 0-0-120°. A range of motion below 90° was considered as knee stiffness [13]. Due to its clinical importance, the extension deficit was recorded and categorized as ≤ 10° or > 10°. Furthermore, the complications deep venous thrombosis (DVT), heterotopic ossifications, and knee instability were recorded based on the latest available radiological or clinical controls within the 6-month follow-up period. Moreover, the need for reoperation of the fracture within 6 months was recorded. This did not include the planned removal of the external fixation apparatus.

Valgus and varus malalignments were measured by the medial proximal tibial angle (MPTA) on antero-posterior radiographs. This was measured by drawing a line in parallel with the tibial joint surface and measuring the medial angle between this line and a line drawn along the long axis of the tibia. The values > 5° from 90 were considered as malalignment [14]. The proximal posterior tibial slope (PPTS) was measured on lateral radiographs with the same method, 8° were subtracted to correct for the posterior tibial tilt, and the values deviating > 5° were judged as misalignment [15]. All measurements were performed on the latest possible radiograph available within the follow-up period. The evaluation of records and radiographs was double checked (HB), analyzed for conformity with patient’s records, and supervised (HS).

### Statistical analysis

Statistical analysis was done using STATA 14.2 (StataCorp LLC, College Station, TX, USA). The unpaired *t* test was used for normally distributed continuous data and the Mann-Whitney *U* test for non-parametric ordinal data. Categorical data were compared using the chi-square test or the Fisher’s exact test, when the sample size of an outcome was less than 5. When 3 or more categorical parameters were included in one analysis, a R by C chi-square test was used. A logistic regression analysis for binary outcomes reporting odds ratios facilitated correction for uneven distributed risk factors (smoking, injury severity score), which was calculated for all outcome parameters. Since both parameters potentially negatively influence the outcome, they were included in one single model. Furthermore, influencing factors for the development of non-unions were analyzed using logistic regression including classification, dislocation, incidence of deep infections and open fractures, and ISS. A *P* value less than 0.05 was considered statistically significant.

## Results

### Epidemiological parameters

**Fig. 1:**
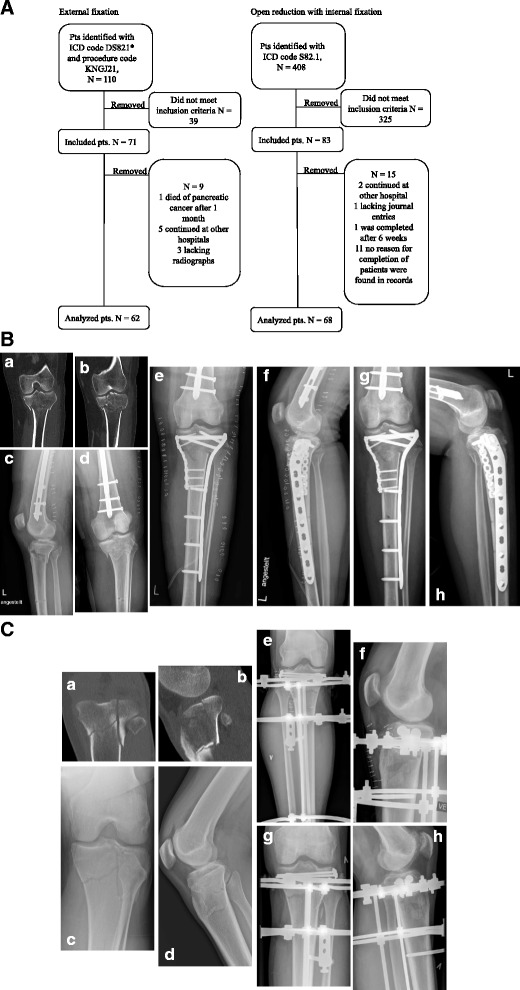
**a** Flowchart. The flowchart indicates how the patients were identified including the reasons for lost follow-ups. **b** Plating. A–D show X-ray and CT scanning of an AO 41 C3 fracture with varus malalignment in a 34-year-old female patient. Considering the distal femur shaft fracture, the injury may be considered as a “floating knee.” E–F show the same fracture fixated with locking plates. G–H demonstrate AP and lateral views 6 weeks later. **c** External fixation. A–D show X-ray and CT scanning of an AO 41 C1 fracture in a 33-year-old male patient. E–F show the same fracture following screw fixation of the articular surface and stabilization with external fixation. G–H demonstrate AP and lateral views 6 weeks later

One hundred ten patients treated with external fixation and 408 with plate fixation were identified. Seventy-one patients fulfilled the inclusion criteria in the external fixation (EF) group; however, 9 patients needed to be excluded because of incomplete records, resulting in a loss-to-follow-up rate of 12.6%. In the plate fixation (PF) group, 83 patients were included. Fifteen needed to be excluded, causing the loss-to-follow-up rate of 18%. The details are documented in Fig. 1. Sixty-two patients were analyzed in the EF and 68 patients in the PF group. Two weeks after the initial operation, one patient with plate fixation was converted to hybrid external fixation due to deep infection and was analyzed on an intention-to-treat basis as a plate fixation treatment. The groups were similar in terms of age, BMI, gender, and ASA score with no significant statistical differences (Table 1). There was a higher proportion of tobacco smokers in the EF group (41.9%) compared to the PF group (20.6%, *p* = .008).

**Table 1:** Patient epidemiology

		External fixation (*N* = 62)	Plate fixation (*N* = 68)	*P* value
Mean age (years)		55.74 ± 14.3	50.44 ± 14.7	.96
Mean BMI		25.56 ± 4.77	25.6 ± 4.27	.447
Gender	Male/female	30/32	36/32	.88
ASA score (%)	ASA 1	21 (33.8)	16 (23.52)	
	ASA 2	29 (46.77)	38 (55.88)	.414
	ASA 3	12 (19.35)	14 (20.58)	
Smoking (%)	Smokers	26 (41.9)	14 (20.6)	.008

The table provides an overview about patient’s epidemiological parameter in both groups

*BMI* body mass index, *ASA* physical status according to the American Society of Anesthesiologists

### Injury parameters

The two treatment groups had a comparable rate of open fractures, similar trauma mechanisms, mean displacement of the fracture fragments, and pre-operative compartment syndromes or incidences of peroneal nerve paresis (Table 2). However, the plate fixation group had a statistically significant higher mean ISS (13.56) compared to the group undergoing external fixation (10.08, *p* = .006). Accordingly, more polytrauma patients with an ISS above 16 were seen in this group (23.5% compared to 8.1%, *p* = .017), and more isolated tibia fractures were found in the EF group (67.7% compared to 50%, *p* = .04). The majority of fractures in both groups were 41 C3 fractures (EF 54.8%, PF 58.8%, *p* = .647). No 41 A-type fractures were treated by external fixation, and the portion in the group with plate fixation was also low (7.4%, *p* = .036). There were no significant differences between the proportions of 41 A2, C1, and C2 fractures (Table 2).

**Table 2:** Injury characteristics

		External fixation (*N* = 62)	Plate fixation (*N* = 68)	*P* value
Open fractures (%)		7 (11.3)	12 (17.6)	.305
AO classification (%)	A2	3 (4.8)	9 (13.2)	.099
	A3	0	5 (7.4)	.036
	C1	9 (14.5)	5 (7.4)	.188
	C2	16 (25.8)	9 (13.2)	.069
	C3	34 (54.8)	40 (58.8)	.647
Trauma mechanism (%)	High energy	47 (75.8)	59 (86.8)	.108
	Low energy	15 (24.2)	9 (13.2)	.108
Mean ISS		10.08 ± 3.074	13.56 ± 8.9	.006
Mean displacement (mm)		12.07 ± 7.38	12.85 ± 8.39	.765
Severity (%)	Monotrauma	42 (67.7)	34 (50)	.04
	Multiple injuries	15 (24.2)	18 (26.5)	.76
	Polytrauma	5 (8.1)	16 (23.5)	.017
Compartment sy. (%)	Pre-operatively	11 (16.2)	6 (9.7)	.272
N. peroneus paresis (%)	Pre-operatively	2 (3.22)	7 (10.29)	.106

The table provides an overview about patient’s injury characteristics in both groups

*ISS* Injury Severity Score, *sy.* syndrome

### Healing and non-union

In both groups, most fractures healed after 3–6 months. However, the distribution of patients in the three groups (healed ≤ 3 months, between 3 and 6 months, non-union) was significantly different (*p* = .0416). Considering the asymmetric distribution, the subgroups of patients with successful healing and with a non-union were analyzed separately. There was a higher portion of patients, who were healed after 3 months in the group undergoing internal fixation. Primarily, the chi-square test failed to show statistical significance (*p* = .057); however, when including the factors smoking behavior and ISS in a logistic regression analysis, the difference between the groups reached statistical significance (*p* = .041, Table 3). Non-unions were seen in 4.8% of EF and in 13.2% of PF cases without a statistically significant difference (*p* = .099).

**Table 3:** Outcome and complications

		External fixation (*N* = 62)	Plate fixation (*N* = 68)	*P* value
Healing *n* (%)	0–3 months	17 (27.4)	27 (39.7)	.041^*^
	3–6 months	42 (67.7)	32 (47.1)	–
Non-union *n* (%)	All fractures	3 (4.8)	9 (13.2)	.099
	C1–3	3 (5)	4 (7.4)	.45
	A2–3	0	5 (35.7)	.324
Median ROM		107.5 ± 19	117.5 ± 20.9	.091
Mean MPTA		88 ± 4.25	88.32 ± 2.09	.677
Mean PPTS		9.3 ± 4.2	10.9 ± 4.5	.084
Extension deficit (%)	1–9°	10 (16.12)	10 (14.7)	.822
	> = 10°	8 (12.9)	10 (14.7)	.766
Knee stiffness (%)		7 (11.29)	9 (13.23)	.736
Varus malalignment (%)		5 (8)	2 (2.9)	.184
PPTS misalignment (%)		13 (20.1)	13 (19.1)	.792
Total misalignment (%)		16 (25.8)	15 (22)	.616
Knee instability (%)		5 (8)	3 (4.41)	.309
Superficial infection (%)		25 (40.4)	2 (2.9)	.000
Deep infection (%)		6 (9.67)	5 (7.35)	.634
Reoperation (%)		8 (12.9)	10 (14.7)	.766
HTO (%)		1 (1.6)	9 (13.2)	.013
Signs of PO		42 (67.8)	34 (50)	.02
PO difference (%)	0	42	45	
	1	10	17	
	2	9	4	.273
	3	1	2	
Mean difference PO		.50 (.80)	.45 (.74)	.933
Compartment sy. (%)	Post-operatively	0	0	
N. peroneus paresis (%)	Post-operatively	3 (4.83)	0	.106
DVT (%)		2 (3.22)	1 (1.47)	.465

The table provides an overview about outcome and complications in both groups

*ROM* range of motion, *MPTA* medial proximal tibial angle, *PPTS* proximal posterior tibial slope, *HTO* heterotopic ossifications, *PO* posttraumatic osteoarthritis, *DVT* deep venous thrombosis, *sy.* syndrome

^*^The value represents the significance level after logistic regression including the uneven distributed parameters ISS and smoking

### Range of motion and malalignment

The range of motion was slightly better in the plate fixation group with a median of 117.5° compared to 107.5°, but this was not found to be statistically significant (*p* = .091). There were no significant differences in the rates of extension deficit, knee instability, and knee stiffness. The mean medial proximal tibial angle, the mean proximal posterior tibial slope, varus/valgus malalignment, and PPTS misalignments were also equal (Table 3). All coronal misalignments seen were varus misalignments (8% EF vs. 2.9% PF, *p* = .184). Two patients had both varus and proximal posterior tibial slope misalignment and were counted as one case in each group in the total misalignment calculation, which showed no significant difference between the groups (EF 25.8% vs PF 22%, *p* = .616).

### Infection and reoperation within 6 months

Superficial infections were seen in 40.4% of the external fixation cases and 2.9% in the plating group, a difference with high statistical significance (*p* = .000). This could be confirmed using a logistic regression analysis (Table 4). However, there was no statistically significant disparity between the rates of deep infection with 9.67% in the external fixation group compared to 7.35% in the plating group (*p* = .634). An overview is provided in Table 3. In two patients from the external and one patient in the plate fixation group, the deep infection became apparent first after 6 months. Despite of this, they were included in the presented calculations. Smoking was not associated with deep infections, which occurred in 6 (6.6%) non-smokers and 5 (12.5%) smokers (*p* = .219). With regard to open fractures, deep infections occurred in 9 (8.1%) closed fractures and 2 (11.76%) open fractures, which did not reach a statistically significant difference (*p* = .50). There was no difference in the reoperation rate after 6 months between the groups with 8% in the EF group and 10% in the PF group (*p* = .766). In the PF group, 4 reoperations were due to lacking signs of healing, which were treated with bone grafting and/or readjustment of the internal fixation. Three were due to deep infection and 2 because of loosening with mechanical irritation. The last operation was an arthroscopic arthrolysis performed because of a limited ROM. In the external fixation, group 4 reoperations were due to deep infection. An arthroscopic arthrolysis due to limited ROM and bone grafting because of lacking healing signs were also carried out. In one patient, further fracture displacement occurred, which was operatively corrected. The last reoperation in this group was due to a superficial infection. This patient had also complaints and discomfort, for which is the reason the wire frame was removed together with local debridement. During the operation, no signs of deep infection were seen, and later, bacterial cultures were negative.

**Table 4:** Logistic regression analyzing outcome criteria corrected for uneven distributed risk factors (smoking, ISS)

Parameter	Odds ratio	*P*	95% confidence interval
Superficial infection	25.70 ± 20.85	0.000	5.24–126.05
Deep infection	1.14 ± 0.76	0.846	0.31–4.21
DTV	2.21 ± 2.76	0.527	0.19–25.64
HTO	0.08 ± 0.089	0.021	0.01–0.69
Reoperation	0.88 ± 0.47	0.808	0.31–2.50
Non-union	0.46 ± 0.34	0.293	0.11–1.95

The table provides an overview about results of a logistic regression analysis for outcome criteria corrected for uneven distributed risk factors (smoking, ISS)

*ISS* injury severity score, *DVT* deep venous thrombosis, *HTO* heterotopic ossifications

### Heterotopic ossification and posttraumatic osteoarthritis

There were more cases of heterotopic ossification in the plating group with 13.2% compared to 1.6% in the external fixation group (*p* = .013). This could be confirmed using a logistic regression analysis (Table 4). There were more signs of posttraumatic osteoarthritis in the EF group (EF 67.8%, PF 50%, *p* = .02); however, this included patients with pre-existing osteoarthritis. When looking purely at the difference between pre-operative and post-operative Kellgren-Lawrence scores, there was no disparity for the incidence of posttraumatic osteoarthritis (Table 3).

### Postoperative compartment syndrome, peroneal nerve paresis, and thrombosis

There were no cases of postoperative compartment syndromes in either group (Table 3). There were 3 postoperative peroneal nerve palsies/paresis in the external fixation group but none in the other group; however, the difference was not statistically significant (*p* = .106). No difference in the rate of deep venous thrombosis (DVT) was seen (EF 3.22%, PF 1.47%, *p* = .465).

### Risk factors for non-unions

ISS and fragment displacement were higher in the group with non-unions (*p* = .0015 and *p* = .0002, respectively, Table 5). Furthermore, the fracture classification had an influence on the development of non-unions. Type A fractures were associated with a higher risk (*p* = .002). Smoking, age, ASA grade, or the presence of an open fracture did not seem to influence the incidence of non-unions (*p* = .22, *p* = .47, *p* = .46, and *p* = .075, respectively, Table 5). There were 3 cases of deep infection in the non-union patients in the plating group but none in the external fixation group, but the incidence of deep infections did not primarily promote the development of non-unions (*p* = .065). The factors classification, dislocation, incidence of deep infections and open fractures, and ISS were included in a logistic regression analysis, which finally identified the independent risk factors for the development of non-unions (Table 6): classification, displacement, and deep infection.

**Table 5:** Epidemiological parameter and injury characteristics in patients with non-unions

		Non-union (*N* = 12)	Union within 6 months (*N* = 118)	*P* value
Mean ISS		16.1 ± 7.95	11.46 ± 6.75	.0015
Mean displacement (mm)		19.49 ± 6.35	11.89 ± 7.79	.0002
AO type A/C fractures (%)		41.7/6.6	58.3/93.4	.002
Smoking (%)		2 (16.6)	38 (32.2)	.222
ASA grade (%)	ASA 1	3 (25)	34 (28.8)	
	ASA 2	5 (41.6)	62 (52.5	.47
	ASA 3	4 (33.3)	22 (18.6)	
Age		50 ± 15.19	53.27 ± 14.72	.46
Deep infection (%)		3 (25)	8 (6.7)	.065
Open fracture (%)		4 (33.3)	15 (12.7)	.075

*ISS* injury severity score, *ASA* physical status according to the American Society of Anesthesiologists

**Table 6:** Logistic regression analyzing the influence of different factors on the development of non-unions

Parameter	Odds ratio	*P*	95% confidence interval
Classification AO type A	10.61 ± 8.61	0.004	2.17–52.02
Dislocation	1.11 ± 0.45	0.011	1.02–1.20
ISS	1.05 ± 0.04	0.149	0.98–1.13
Deep infection	7.72 ± 6.99	0.024	1.31–45.58
Open fractures	2.08 ± 1.65	0.355	0.44–9.88

*ISS* injury severity score

## Discussion

The main finding of this study is that external fixation of proximal tibia fractures is associated with a higher rate of superficial infections, but has a lower incidence of heterotopic ossifications compared to internal stabilization using locking plates. Furthermore, bony consolidation occurred slightly earlier in the plate fixation group. The likelihood to develop a non-union significantly depended on fracture displacement and classification and was associated with deep infections.

In agreement with our data regarding earlier healing following internal stabilization, Krupp et al. described a decreased time to union after plate fixation of bicondylar tibia fractures reporting an average of 5.9 months compared to 7.4 months for external fixation [13]. Although a recent meta-analysis comparing both treatment methods for tibial plateau fractures found no significant difference regarding the time to union [7], the recorded data document a similar tendency (17.73 ± 4.87 after external fixation vs 15.64 ± 4.36 weeks following plate fixation). This study included 22 case series and a retrospective cohort study, indicating the lack of direct comparing trials and a low summary evidence level. Similarly, a case series published by Cole et al. investigating the time to union in proximal tibia fractures following internal fixation using the Less Invasive Stabilization System (LISS) reported a time to full weight bearing without pain after an average of 12.6 weeks [11]. Clinically, the reason might also be associated to a more courageous decision regarding weight bearing, when an internal stabilizer is in place to support the healed bone. The incidences of non-union were similar in both groups and were associated with fracture displacement and classification, and the occurrence of deep infections. This is supported by the literature, showing no significant differences in the rates of non-unions [7, 13, 14]. A meta-analysis by Bhandari et al., examining extra-articular tibial fractures, described a trend towards higher non-union rates after external fixation; however, this study questioned its conclusions itself because of its low evidence grade [16]. Our reported rates of non-union are in line with earlier studies, reporting a rate of non-unions up to 13% [5–7, 11, 13, 15, 17, 18]. The fact that superficial infections are more common following external fixation corresponds well with the literature. A meta-analysis by Metcalfe et al., comparing 6 retrospective cohort studies, reported an odds ratio of 2.96, when external was compared to internal fixation with plating [19]. Case series, investigating complications in fractures treated by Ilizarov frame fixation published by Keitgthley et al. and El-Sayed et al., showed superficial infection rates of 51.3 and 41.8%, respectively, which is similar to the 40.4% rate seen in this study [5, 20]. Like our results, heterotopic ossifications occurred also more frequently in other series following plate fixation compared to external fixation [7]. However, our reported rates were higher than other incidences, stating an average of only 1.23% [7]. This might be caused by a subjective evaluation and lacking standards. A study published by Krupp et al. reported higher rates ranging at 7.14% after plate fixation, which also was higher than following external fixation [13]. Considering the results for joint mobility, the statistically significant difference seemed not to limit function. Extensive soft tissue and bone damage often accompany high-energy injuries of proximal tibia fractures, increasing the likelihood of infectious complications and affecting the treatment algorithm [3, 18]. Indeed, deep infections are a severe problem and have often been reported as relatively frequent following plate fixation in proximal tibia fractures. A RCT by the Canadian Orthopedic Trauma Society reported 17%, which was often used to argue for external fixation [13, 21]. However, the study by Krupp et al. describes a higher deep infection rate following external fixation ranging at 13% [13]. Besides this, the meta-analyses by Yu et al., Metcalfe et al., and Bhandari et al. support the results found in our study and reported comparable rates for both methods [7, 13, 16, 19]. The data described in the literature support the concept that an operative method should be practiced by a surgeon and a team, which is used to do this. This is probably sometimes more important than the method itself and might explain the large discrepancies of published data. All steps from indication to aftercare require a certain competence, which is connected to experience and daily practice. Therefore, this study had a two-center-two-method design, considering the necessity of routine for each type of treatment. Furthermore, this study presents to our knowledge the highest number of patients in one trial published to date. Corresponding with our data, the range of motion and knee stiffness has been reported to be similar after fixation with one of the two examined methods [7, 19, 21]. However, Krupp et al. reported a trend towards higher rates of extension deficits and knee stiffness following external fixation, but this was not conclusive [13]. Similarly, Conserva et al. reported problems in 7.3% of tibia fractures that underwent framing compared to 0% treated by plate fixation [22]. Unfortunately, this study included also type Schatzker IV and V fractures; so, it remains unclear how applicable this observation is regarding fractures with a complete metaphyseal component. Although the incidences of deep venous thrombosis, reoperations, compartment syndromes, and peroneal nerve paresis are decisive for the single affected patient, they are overall not very frequent and were observed in both groups with the same frequency. This conforms with the data published [7, 19]. Metaphyseal-diaphyseal misalignments have been identified as one of the decisive parameters determining the clinical outcome. Corresponding with other studies, no significant differences between the two groups could be found in the presented study [14, 16, 21]. However, as described in the meta-analysis by Yu et al. [7], it is important to define how the measurements were done methodologically. This might also explain the differences in various studies reporting rates ranging for both groups between 3.3 and 43% [13, 14, 21]. Although this study focused on the stabilization of the metaphyseal component of the proximal tibia fractures, the incidence of posttraumatic osteoarthritis has been included since the correct alignment is not only decisive for the clinical outcome [23] but also for the development of osteoarthritis. Interestingly, the analysis by Yu et al. [7] indicated a higher percentage with arthritis in the external fixation group. However, other studies found no significant difference. One reason, which needs certainly to be considered in this background, is the follow-up time. Only the study by Jansen et al. reported a mean follow-up period of 67 months; usually, the time frame was between 6 and 24 months [13, 17, 19, 21, 24]. However, the posttraumatic osteoarthritis does not seem to be decisive for short-term patient reported outcome measures [25], and might be more important, when long-term results are compared. In contrast, knee instability immediately affects the clinical satisfaction and has been reported to be more frequent following external fixation compared to plating [7]. Our data did not indicate a difference just as described by the case series published by El-Sayed et al. [5], investigating Schatzker VI fractures, and the study by Krupp et al., which reported rates between 3.6 and 3.3%, respectively [13].

Several epidemiological parameters are known confounders influencing our primary outcome, the bone healing. The data confirm that initial displacement and the incidence of deep infections are independent risk factors for the development of non-unions. However, both incidences were equally distributed between our groups. In contrast, frequencies of both smoking behavior and the injury severity score were differently distributed between patients undergoing either external fixation or plating. Considering their potential influence on healing [26–28], these factors were included in a logistic regression analysis, which finally resulted in the conclusion that the healing time following plating was slightly shorter.

Limitations of the study are the sample size and the retrospective study design, which encounter the risk to miss significances and incomplete documentation. Moreover, rehabilitation and physiotherapy were assumed to follow standard guidelines, but this was partially not individually documented. Some outcome parameters are based on measurements in X-rays; however, the technical adjustments such as size and overview differed between the various pictures. To minimize the resulting bias, two examiners have contributed to the evaluation being especially critical when the values ranged around the set cutting values. Furthermore, the follow-up period and the outcome parameters were limited, lacking patient-related outcome measures.

Naturally, these limitations could be avoided when applying a different study design. Considering the limited case load of these injuries, a register or a multicenter setup would be preferable.

## Conclusions

Our results indicate that healing of proximal tibia fractures with a complete metaphyseal component occurs slightly earlier with plate fixation and that superficial infections after external fixation and heterotopic ossification following plate fixation are relevant complications, which should be encountered. Therefore, these aspects can be taken into consideration, when a certain fixation method is chosen. However, since the detected differences are very likely without consequences for patient’s outcome exceeding a 6-month period, the method of choice should mainly depend on the local experience including the established treatment infrastructure.

## Additional file

Additional file 1: The file contains the anonymized raw data. (XLSX 27 kb)

## Abbreviations

ASA: Physical status according to the American Society of Anesthesiologists
BMI: Body mass index
DVT: Deep venous thrombosis
EF: External fixation
HTO: Heterotopic ossifications
ISS: Injury Severity Score
MPTA: Proximal tibial angle
OR: Odds ratio
PF: Plate fixation
PO: Posttraumatic osteoarthritis
PPTS: Proximal posterior tibial slope
ROM: Range of motion
